# Expanded detection and impact of *BAP1* alterations in cancer

**DOI:** 10.1093/narcan/zcae045

**Published:** 2024-11-15

**Authors:** Ian R Sturgill, Jesse R Raab, Katherine A Hoadley

**Affiliations:** Bioinformatics and Computational Biology Curriculum, Department of Genetics, University of North Carolina at Chapel Hill, 116 Manning Drive, Chapel Hill, NC 27599, USA; Department of Genetics, Lineberger Comprehensive Cancer Center, University of North Carolina at Chapel Hill, 116 Manning Drive, Chapel Hill, NC 27599, USA; Department of Genetics, Lineberger Comprehensive Cancer Center, University of North Carolina at Chapel Hill, 116 Manning Drive, Chapel Hill, NC 27599, USA

## Abstract

Aberrant expression of the *BAP1* (*BRCA* associated protein 1) tumor suppressor gene is a prominent risk factor for several tumor types and is important in tumor evolution and progression. Here we performed integrated multi-omics analyses using data from The Cancer Genome Atlas for 33 cancer types and over 10 000 individuals to identify alterations leading to *BAP1* disruption. We combined existing variant calls and new calls derived from a *de novo* local realignment pipeline across multiple independent variant callers, increasing somatic variant detection by 41% from 182 to 257, including 11 indels ≥40 bp. The expanded detection of mutations highlights the power of new tools to uncover longer indels and impactful mutations. We developed an expression-based *BAP1* activity score and identified a transcriptional profile associated with *BAP1* disruption in cancer. *BAP1* has been proposed to play a critical role in controlling tumor plasticity and normal cell fate. Leveraging human and mouse liver datasets, *BAP1* loss in normal cells resulted in lower *BAP1* activity scores and lower scores were associated with a less-differentiated phenotype in embryonic cells. Together, our expanded *BAP1* mutant samples revealed a transcriptional signature in cancer cells, supporting *BAP1*’s influences on cellular plasticity and cell identity maintenance.

## Graphical Abstract

**Graphical Abstract:**
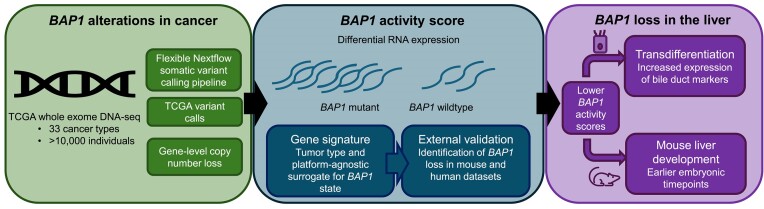

## Introduction

*BRCA* associated protein 1 (*BAP1*) has been identified as a tumor suppressor gene with activity across a broad range of biological processes (1–3), including DNA damage repair (1,4,5), cell cycle and cell proliferation (1,6–8), and apoptosis (8–10). *BAP1* mutations promote development of cancer across multiple tissue types and are associated with differential survival outcomes, for example worse outcomes in uveal melanoma, kidney renal clear cell carcinoma and hepatocellular carcinoma, but improved outcomes in mesothelioma (11–18).

*BAP1* mutations are most prevalent in uveal melanoma, mesothelioma and renal cell carcinoma (1,19). Mutations occur very infrequently in other cancer types, often leading to exclusion from consideration due to underpowered analyses. However, among the cancer types that have been studied, consequences of *BAP1* loss are pleiotropic and further tissue-specific differences have been documented (20). It is therefore reasonable to assume that there is tissue-specific context that has not yet been systematically explored, presenting an opportunity to expand our understanding of the role of *BAP1* in cancer—particularly if we can incorporate additional cancer types into a larger set of analyses of *BAP1* alteration.

Although *BAP1* mutations have been studied in a small subset of cancer types (1,13–16,19), there has been limited incorporation of other types of alterations that can lead to *BAP1* loss. In mesothelioma, for example, there has historically been a substantial underestimation of *BAP1* mutations; Nasu *et al.* and Yoshikawa *et al.*, among others, addressed this through integration of multiple individual technical approaches (21–24). Consistent with these results, by accounting for copy number loss and long indels, The Cancer Genome Atlas (TCGA) demonstrated an increase of 40% of alterations over single-nucleotide variants alone, leading to decreased *BAP1* expression among patients with mesothelioma represented in the TCGA dataset (25). This suggests that many prior studies that focused more on single-nucleotide variants have missed important subsets of *BAP1*-altered samples, thereby impacting interpretation of the role of *BAP1*. Here, we account for these additional types of alterations across a broader set of cancer types represented in TCGA to better characterize functional consequences of *BAP1* alteration in cancer. We previously demonstrated that *BAP1* loss characterized a cholangiocarcinoma-like (CHOL-like) expression-based subset of liver hepatocellular carcinomas and hypothesized that CHOL-like liver tumors represented *BAP1*-driven changes resembling a transdifferentiated cell phenotype (13). To understand the specific impact of *BAP1* loss in both cancer and normal biology, we developed an RNA expression-based *BAP1* activity score and validated this score in external human and mouse liver datasets. We linked this activity score to embryonic development in murine liver supporting a role for *BAP1* driving changes in phenotypic plasticity and cell states.

## Materials and methods

### Somatic mutation calling workflow with *de novo* local realignment

Exon coverage in whole exome DNA sequencing BAM (binary alignment map) files from TCGA varies across tumor types, but coverage generally ranges from 20× to 100×. In this study, 10 414 pairs of tumor and matched-normal aligned BAM slice files of the *BAP1* locus and 100-kb flanking regions from 33 cancer types were downloaded from TCGA Genomic Data Commons (GDC)’s harmonized hg38 mapped data using the command line GDC Data Transfer Tool v.1.6.1. The specific genomic region was hg38 chr3:52300003–52511030. Usage of BAM slices rather than full whole exome BAM files reduced pan-cancer (*n* = 22 286) storage requirements by 99.97% from 447.4 TB down to 120.3 GB. Individual BAM slices were then processed through a Nextflow DSL 2 pipeline for somatic mutation calling that leveraged parallelization to substantially reduce analysis time relative to an unoptimized workflow. Briefly, slices were indexed using Samtools (version 1.9) (26). Reads were realigned to the hg38 human reference genome (GRCh38.d1.vd1) using the ABRA2 (version 2.24-0) *de novo* local realigner with --undup and --no-edge-ci parameter flags (27). Somatic mutations were called using the Strelka2 somatic workflow (version 2.9.10) and Cadabra indel (version 2.24-0) variant callers after marking duplicate reads using bammarkduplicates from biobambam (version 2.0.87) (27–29). Raw variant call format output files were converted to mutation annotation format (MAF) using vcf2maf (Cyriac Kandoth, 2021, mskcc/vcf2maf: vcf2maf v.1.6.21) and VEP (cache version 103) (30).

### Filtering, manual review and characterization of somatic variants

Variant calls were initially filtered based on individual variant caller quality tags (i.e. FILTER == PASS). These variant calls were merged with calls from the GDC’s internal mutation calling pipeline consisting of four variant callers: MuSE, MuTect2, VarScan2 and Pindel. Variants were further filtered to those that had variant allele frequency ≥0.2 and mutant allele read count (t_alt_count) ≥2 followed by manual review using the Integrative Genomics Viewer (IGV) to further assess read support strength and evidence of any other confounding factors such as alignment at low-complexity regions and variant bases captured only by ends of reads (31). Current variant calls for *BAP1* were then compared to historical calls generated for TCGA in the MC3 dataset and characterized by their somatic MAF variant classification (32). Chromosomal start locations of mutations were plotted, together with variant classification information, on a lollipop plot created using cBioPortal’s MutationMapper web tool (33,34). Protein-level domain annotations and visualization were approximated using data from UniProt (35).

### Definition of copy number loss

Estimates of focal gene-level copy number for *BAP1* and other genes were downloaded from the GDC, which used the hg38 ABSOLUTE LiftOver workflow to provide per-gene integer value estimations of gene copy numbers for each sample (36,37). Samples with gene-level copy number of <2 corresponded to copy number loss. Segment widths of samples with copy number loss were computed from segment start and end loci from masked copy number segment files from the GDC, which provided estimates of genomic windows of segments containing the *BAP1* gene locus using the DNAcopy workflow. Plots of segments in relation to the chromosome were generated using plotgardener (version 1.10.2) (38).

### Differential messenger RNA expression and gene set enrichment analyses

Per-sample hg38 STAR (Spliced Transcripts Alignment to a Reference)-aligned unstranded counts files were downloaded from the GDC (39). Where appropriate, we adjusted for additional sources of variation (tumor purity, histological subtype and/or previously defined molecular subtype) in our model design in a cancer type-specific manner (Supplementary Table S3B). Samples without sufficient subtype annotations and samples without tumor RNA sequencing data available were excluded from analyses. Sample groups were then tested for differential gene expression using the DESeq2 R package (version 1.44.0) (40). Briefly, we performed Wald testing with independent hypothesis weighting provided by the IHW R package (version 1.32.0) (41).

Initial results were then subjected to adaptive shrinkage estimation using the apeglm R package (version 1.26.1) (42). Genes were annotated using biomaRt (version 2.60.1) via the annotables R package (Stephen Turner, 2023, version 0.2.0) (43,44). Gene set and pathway analyses were conducted using the fgsea R package (Gennady Korotkevich *et al.*, 2021, version 1.30.0) and data from the C8 gene sets from the MSigDB database (45). Volcano plots were generated using the EnhancedVolcano R package (Kevin Blighe *et al.*, 2018, version 1.22.0) and heatmaps were generated using the ComplexHeatmap R package (version 2.20.0) (46).

### Gene set and *BAP1* activity scores

*BAP1* activity scores were computed based on a gene set of differentially expressed genes between altered-with-mutation (Mut and Mut + CN) and unaltered tumors for five cancer types: CHOL, kidney renal clear cell carcinoma (KIRC), liver hepatocellular carcinoma (LIHC), mesothelioma (MESO) and uveal melanoma (UVM). We generated a weighted activity score signature by taking the sum of the upregulated *BAP1* mutant-associated genes multiplied by −1 and the downregulated genes multiplied by +1 so that low scores reflect low *BAP1* activity. This score was comparable with the rank-based singscore method (*r*^2^= 0.88, singscore R package version 1.24.0) (47). We used the mclust (version 6.1.1) R package with default parameters to perform model-based clustering of activity scores and classify samples as mutant-like or wildtype-like (48). This scoring was then applied to all samples in TCGA. For the bile duct gene signature, gene sets BILE_DUCT_CELLS_1–4 from the MSigDB C8 cell type signature database were combined (45). Bile duct signature scores were computed as *z*-scores of median-centered, log_2_-transformed counts of genes from the combined gene set.

### Survival analysis

Curated survival data from (49) were downloaded from the TCGA PanCanAtlas Publications webpage (see the ‘Data availability’ section). Late-stage tumors classified as any stage 4 were excluded from downstream analyses. Tumors classified as any stage 0–2 were reclassified as low stage and tumors classified as any stage 3 were reclassified as high stage. We implemented a maximum progression-free interval (PFI) cutoff based on median within-tumor-type follow-up times to account for large differences in patient follow-up. Tumors were stratified based on mutant-like versus wildtype-like classification. Kaplan–Meier plots and corresponding risk tables were generated using the survival (version 3.7-0) and ggsurvfit (version 1.1.0).

### Statistical analysis

Statistical analysis was performed using functions included in R version 4.4.1. For comparisons across groups of interest, we generally employed pairwise two-sided *t*-tests with Bonferroni adjustment or pairwise two-sided Mann–Whitney *U* tests with continuity correction and Bonferroni adjustment. Comparison of somatic variant locations across the gene length was done with a two-sided Fisher’s exact test. Differential expression analysis results were reported as *P*-values adjusted using the Benjamini–Hochberg (BH) procedure. To explore relationships between variables of interest, we used Spearman correlation and linear regression with Wherry adjusted *r*^2^. For Kaplan–Meier survival curves, 95% confidence intervals are shown along with *P*-values derived from the log rank test from univariate analyses. Specific statistical methods are listed alongside their corresponding results.

## Results

### *BAP1* somatic mutations in TCGA are underestimated and are predominantly deleterious

**Figure 1.**
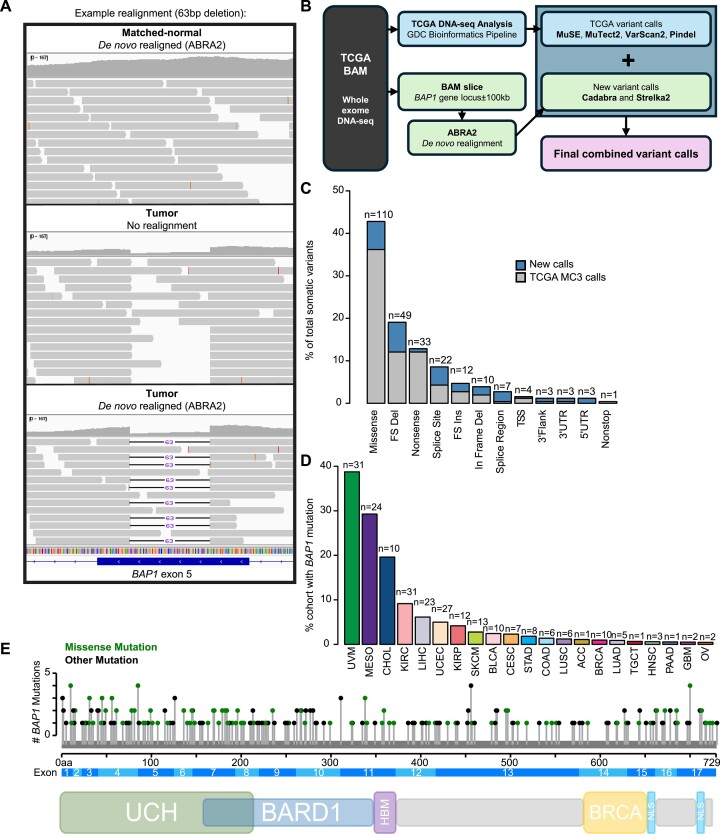
Prevalence and characterization of *BAP1* mutations in TCGA pan-cancer tumor types. (**A**) Example *de novo* realignment with ABRA2 to the hg38 reference genome for a 63-bp indel detected in an LIHC tumor sample (TCGA-FV-A3I0) with visualization in IGV. (**B**) Schematic of the variant calling workflow and combination with TCGA variant calls. (**C**) Distribution of *BAP1* somatic variant types (total = 257 variants). Additional new mutations detected in the present workflow combined with updated TCGA GDC variant calls and prior TCGA MC3 mutation calls are indicated as upper and lower segments of bars, respectively. (**D**) Frequency of *BAP1* mutant tumors by TCGA tumor type. (**E**) Lollipop plot of *BAP1* somatic variant locations across the length of the gene, created using the cBioPortal MutationMapper (33,34). Bottom: protein-level schematic of the major domains of interest, approximated using data from (35). UCH: ubiquitin carboxy-terminal hydrolase; BARD1: *BARD1* binding domain; HBM: *HCF* binding motif; BRCA1: *BRCA1* binding domain; NLS: nuclear localization signal.

To determine the extent of underestimation of *BAP1* mutations in TCGA and add occurrences of larger indels to existing mutation calls, we aligned BAM slice files containing the *BAP1* locus and 100-kb flanking regions to the hg38 genome using a *de novo* local realignment approach with ABRA2 and two variant callers: Cadabra and Strelka2. These variant calls were merged with updated TCGA GDC calls that were also aligned to the hg38 genome and generated from four independent variant callers: MuSE, MuTect2, VarScan2 and Pindel. Out of 10 414 samples and 33 cancer types, a total of 1337 non-synonymous, non-intronic variants (1329 somatic and 8 germline) were detected in 988 individuals across the two updated hg38-aligned approaches, including 14 indels ≥40 bp in length (Supplementary Table S2A). We conducted further review of variants to select those with greater support (tumor variant allele frequency ≥0.2, tumor alternate allele read count ≥2 and manual review of read alignment in IGV), resulting in a reduction from 1337 to 265 variants in 233 individuals (2.2%) across TCGA cancer types, including 11 larger indels ≥40 bp that were not previously detected (Figure 1A and B, and Supplementary Table S2C).

We compared somatic variants (257/265 total variants) to an earlier iteration of variant calling used to generate the TCGA MC3 dataset, which included alignment to the hg19 genome and seven callers: MuTect, Varscan2, Indelocator, Pindel, SomaticSniper, RADIA and MuSE (32). This earlier pipeline resulted in the detection of 251 variants from 224 individuals, of which 190 variants from 170 individuals similarly passed simple call quality filtering, none of which were ≥40 bp (Supplementary Figure S1 and Supplementary Table S2B and D). Our approach yielded an additional 77 unique somatic variant calls, representing a 41% increase over the prior MC3 calls (Figure 1C). All MC3 variants and mutant samples were captured in the new approach, except one variant, a 5 bp deletion in KIRP sample TCGA-2Z-A9JD (Supplementary Tables S2C and D). However, our realignment approach detected two single-nucleotide variants (deletion and missense) in the similar region, suggesting a potential difference in hg19 versus hg38 alignment and downstream variant calling.

The majority of *BAP1* somatic variants were missense mutations (110/257, 42.8%), followed by deleterious nonsense and frameshift combined events (94/257, 36.6%; Figure 1C and Supplementary Table S2C). Of the cancer types represented in TCGA, 21/33 (63.6%) have at least one *BAP1* mutant sample (Figure 1D). Six tumor types had >5% mutation frequency: CHOL, KIRC, LIHC, MESO, uterine corpus endometrial carcinoma (UCEC) and UVM (Supplementary Table S3A).

Somatic variants were broadly distributed across the length of the gene with no significant difference in protein-level domain representation for missense versus other mutation types (two-sided Fisher’s exact test *P*= 0.1) (Figure 1E and Supplementary Table S2C). We observed that 161/257 (62.6%) variants occurred at an annotated functional domain with 110/257 (42.8%) occurring at the catalytic UCH domain (Figure 1E and Supplementary Table S2C). Due to relatively small numbers of variants, we were insufficiently powered to further explore potential differences in impacts to specific functional domains.

### Copy number alterations constitute a major mechanism of *BAP1* loss across cancer types

**Figure 2.**
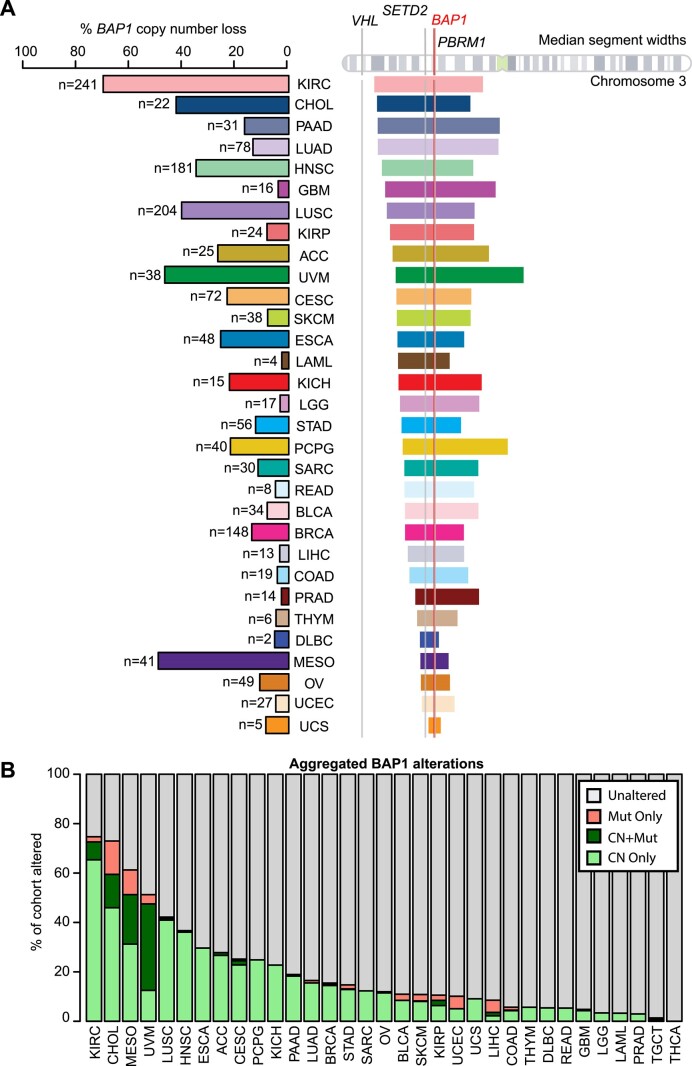
Copy number loss of *BAP1* is frequent in cancer. (**A**) Left: percent and number of samples with *BAP1* gene-level copy number loss for each cancer tumor type. Right: representations of median segment widths of copy number loss in samples with *BAP1* gene-level copy number loss aligned against chromosome 3, which contains the indicated *BAP1* gene locus. The locations of other commonly altered driver genes in cancer (*VHL*, *SETD2* and *PBRM1*) are also indicated. (**B**) Percentage of each tumor type altered for *BAP1*, colored by specific alteration type. Mut Only: mutation only; CN + Mut: co-occurring gene-level copy number loss and mutation; CN Only: gene-level copy number loss only.

To assess the contribution of copy number alterations to a *BAP1* loss phenotype, we examined gene-level copy number estimates of the *BAP1* gene. *BAP1* copy number loss was observed in 1547 samples and estimated at single copy loss in 99% of those samples (Supplementary Table S3A and B). Gene-level copy number loss occurs frequently (>30% of samples) in KIRC, UVM, CHOL, MESO, lung squamous cell carcinoma (LUSC), and head and neck squamous cancer (HNSC) (Figure 2A and Supplementary Table S3A).

We also considered the copy number segment widths around *BAP1* to determine whether the loss is focal or arm level. For many cancer types, the loss is arm level, spanning large regions of the ∼90 Mb chromosome 3p arm on which *BAP1* is located and including regions corresponding to over 1000 annotated genes. There are other commonly lost tumor suppressors on this chromosome (*VHL*, *SETD2* and *PBRM1*). We looked at the width of the deletion segment by tumor type and found variation by tumor type. For example, KIRC [median 60.7 Mb, interquartile range (IQR) 33.6–82.3 Mb], LUAD (lung adenocarcinoma, median 63.0 Mb, IQR 34.6–87.8 Mb) and PCPG (pheochromocytoma and paraganglioma, median 40.8 Mb, IQR 12.3–89.2 Mb) have very large segment widths of loss (Supplementary Table S3B). In contrast, MESO (median 8.3 Mb, IQR 0.2–29.2 Mb), OV (ovarian serous cystadenocarcinoma, median 9.5 Mb, IQR 4.7–19.4 Mb) and UCEC (median 7.2 Mb, IQR 4.6–15.6 Mb) have relatively more focal *BAP1* copy number loss, impacting an estimated 200–360 genes.

Combining copy number and mutation data, 1679 samples from 32 tumor types were considered altered (Figure 2B and Supplementary Table S3A and B). Alterations were predominantly copy number driven (1547/1679, 92.1%). Mutations were less frequent; they occurred alone in 7.9% (132/1679) of altered samples and co-occurred with estimated single-copy copy number loss in 6% (101/1679). Regardless of tumor purity, mean variant allele frequency is 0.46 ± 0.21 (standard deviation). Variant allele frequency is higher in samples with both mutations and copy number loss compared to mutations alone (two-sided Mann–Whitney *U* test with continuity correction *P*= 0.002; Supplementary Figure S2A). Variant allele frequency and tumor purity have a moderate positive correlation (linear regression Wherry adjusted *r*^2^= 0.35; Supplementary Figure S2B), suggesting that these mutations are less likely to be subclonal. The most highly altered cancer types for *BAP1* are KIRC, CHOL, UVM, MESO, LUSC and HNSC—all with >30% altered samples within their tumor types (Figure 2B and Supplementary Table S3A and B). Among altered samples, there is also substantial variability in the mechanism of alteration. In KIRC and HNSC, alterations are primarily driven by instances of copy number loss. However, in CHOL, UVM, MESO and LIHC, mutations appear to play a much larger role. All alteration types, regardless of cancer type, resulted in lower RNA-level expression of *BAP1* than unaltered samples (two-sided pairwise Mann–Whitney *U* test with continuity correction *P*< 0.01 for each alteration type; Supplementary Figure S3 and Supplementary Table S3B).

### Expression-based signatures identify a distinct *BAP1* mutant-like subset

**Figure 3.**
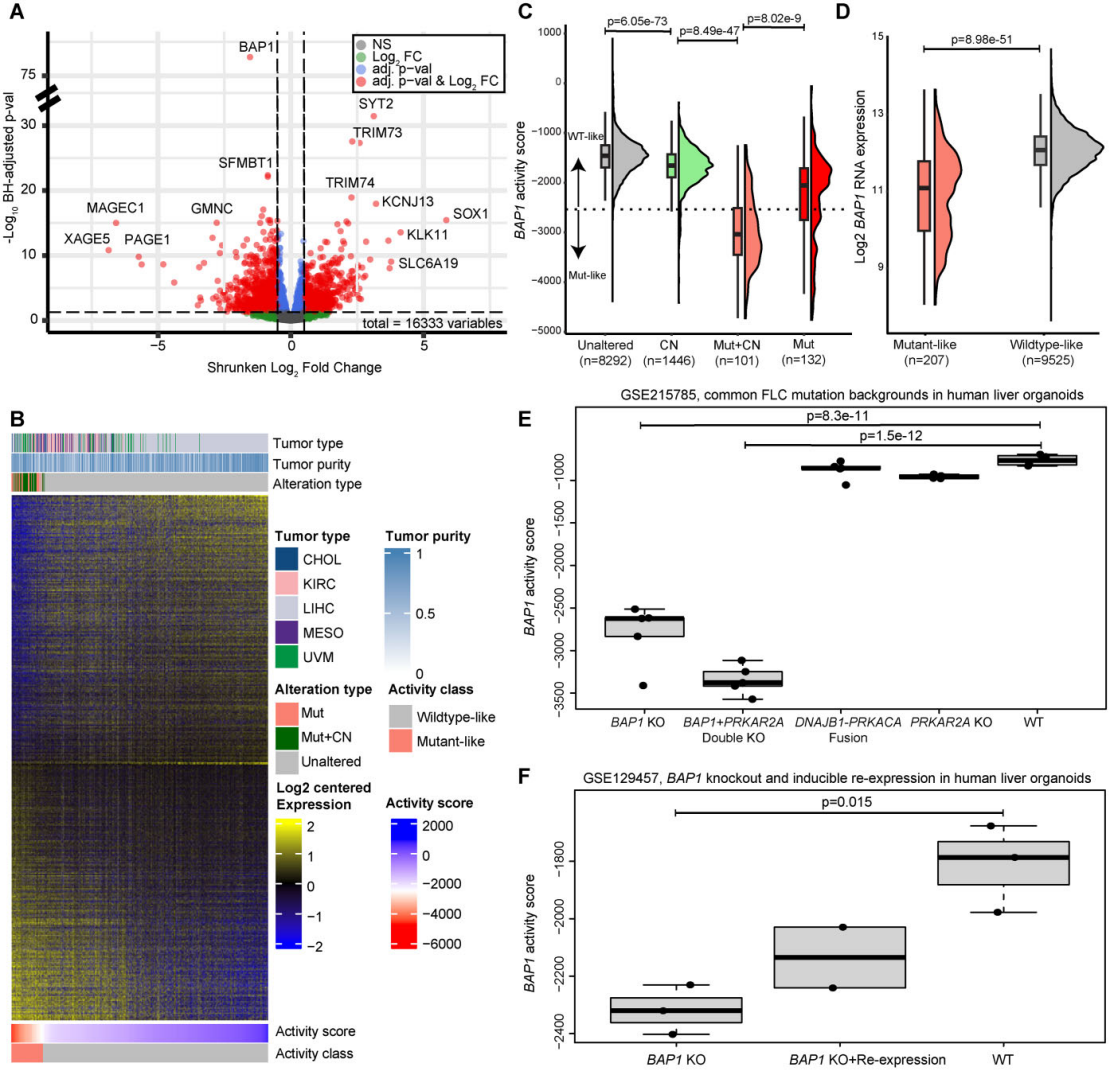
*BAP1* activity scores capture *BAP1*-driven changes in gene expression across multiple independent datasets. (**A**) Volcano plot of *BAP1* differentially expressed genes between *BAP1*mutant and unaltered tumors from TCGA CHOL, KIRC, LIHC, MESO and UVM tumor types. Adjusted *P*-values are derived in DESeq2 using the BH procedure. Log_2_ fold change values were shrunken using the apeglm R package. Abbreviations: adj. p-val & Log_2_ FC: adjusted *P*-value <0.05 and absolute log_2_ fold change ≥1.5; adj p-val: adjusted *P*-value <0.05; Log_2_ FC: absolute log_2_ fold change ≥1.5; NS: not significant. (**B**) Heatmap of differentially expressed genes between *BAP1* mutant and unaltered tumors. Mut: mutation only; Mut + CN: mutation and gene-level copy number loss. (**C**) TCGA pan-cancer activity scores across the *BAP1* alteration types. The dotted line represents the threshold delineating wildtype-like and mutant-like classification. CN: copy number loss only; Mut + CN: mutation and gene-level copy number loss; Mut: mutation only. *P*-values are derived from pairwise two-sided Mann–Whitney *U* tests with continuity correction and Bonferroni adjustment. (**D**) TCGA pan-cancer comparison of *BAP1* RNA expression in wildtype-like and mutant-like tumors. *P*-values are derived from two-sided Mann–Whitney *U* tests with continuity correction and Bonferroni adjustment. (**E**) Comparison of *BAP1* activity scores for a dataset of human liver organoids (GSE215785) with experimentally produced common fibrolamellar carcinoma mutation backgrounds. *BAP1* KO: *BAP1* single knockout; *BAP1*+ *PRKAR2A*: *BAP1* and *PRKAR2A* double knockout; *PRKAR2A* KO: *PRKAR2A* single knockout; WT: wildtype. *P*-values are derived from pairwise two-sided *t*-test with Bonferroni adjustment. (**F**) Comparison of *BAP1* activity scores for a dataset of human liver organoids (GSE129457) with conditional *BAP1* knockout. *BAP1* KO: *BAP1* knockout; *BAP1* KO + Re-expression: *BAP1* knockout with 24-h re-expression; WT: wildtype. *P*-values are derived from pairwise two-sided *t*-test with Bonferroni adjustment.

To better understand the tissue- and context-specific consequences of *BAP1* alteration, we examined differential gene expression across samples with a *BAP1* mutation (*BAP1* mutation and *BAP1* mutation plus copy number loss) and those without any alteration (no mutation or copy number loss) by tumor type among five tumor types with >5 *BAP1* mutant samples: CHOL, KIRC, LIHC, MESO and UVM. We reasoned that comparing *BAP1* mutant to unaltered samples would give the most accurate *BAP1*-dependent gene expression changes. We adjusted for additional sources of known variation, such as tumor purity and subtype in our model designs in a cancer-specific manner (Supplementary Table S3B). Of 16 333 expressed protein-coding genes tested in this manner, 3966 (24.3%) were significantly changed at an adjusted *P*-value <0.05 (Figure 3A and Supplementary Table S4A and B).

Using this set of differentially expressed genes, we computed per-sample *BAP1* mutation signature scores and classified tumors as being more mutant-like or wildtype-like (see the ‘Materials and methods’ section). We ordered tumors by their respective scores, revealing a distinct pattern of gene expression among mutant-like tumors (Figure 3B). Among the five tumor types in this analysis, 57/59 (96.6%) of mutant tumors were classified as mutant-like in this manner. We then applied this mutation signature scoring method to the rest of the TCGA dataset, where 117/233 (50.2%) of mutant tumors were classified as mutant-like, with mutant tumors having lower activity scores and tumors with *BAP1* mutation plus copy number loss having the lowest scores (Figure 3C). This score did not strongly correlate with *BAP1* gene expression (Supplementary Figure S4A). We also determined the impact of other nearby 3p tumor suppressors (*VHL*, *PBRM1* and *SETD2*) and found that their copy number loss was not significantly associated with the BAP1 activity score (Supplementary Figure S4B). We assessed RNA-level expression of *BAP1* based on *BAP1* activity score and classification and confirmed that mutant-like tumors had lower expression, consistent with a signature driven by *BAP1* loss (Figure 3D and Supplementary Figure S5).

To further verify that our *BAP1* activity signature effectively captures a transcriptional profile shift associated with *BAP1* loss, we leveraged two external datasets of *BAP1* knockout in human liver organoids. In the first dataset (GSE215785), common fibrolamellar carcinoma mutant backgrounds were generated, including *BAP1* single-knockout and *BAP1*/*PRKAR2A* double-knockout organoids (50). Both single- and double-knockout organoids had significantly lower *BAP1* activity scores relative to wildtype (pairwise *t*-test with Bonferroni correction *P*= 8.3e−11 and *P*= 1.5e−12, respectively; Figure 3E), but no difference was observed in the samples with the classic *DNAJB1–PRKACA* fusion or *PRKACA* knockout, which is the known major driver of that subset of liver cancer. In the second dataset (GSE129457), *BAP1* knockout organoid models were generated both with and without *BAP1* re-expression (51). Consistent with GSE215785 data in Figure 3E, we also observed reduced *BAP1* activity scores among *BAP1* knockout organoids relative to wildtype (pairwise *t*-test *P*= 0.015; Figure 3F). Re-expression of *BAP1* in the knockout organoid led to increased activity scores relative to knockout (Figure 3F). We used curated survival data from the TCGA Clinical Data Resource to perform tumor-specific survival analyses stratified by *BAP1* activity class and noted improved progression-free survival outcomes for wildtype-like UVM and KIRC tumors (Supplementary Figure S6) (49). We did not have adequate long-term follow-up and were otherwise underpowered to detect differential survival for other tumor types.

### *BAP1* alteration characterizes a molecularly distinct subset of hepatocellular carcinomas

**Figure 4.**
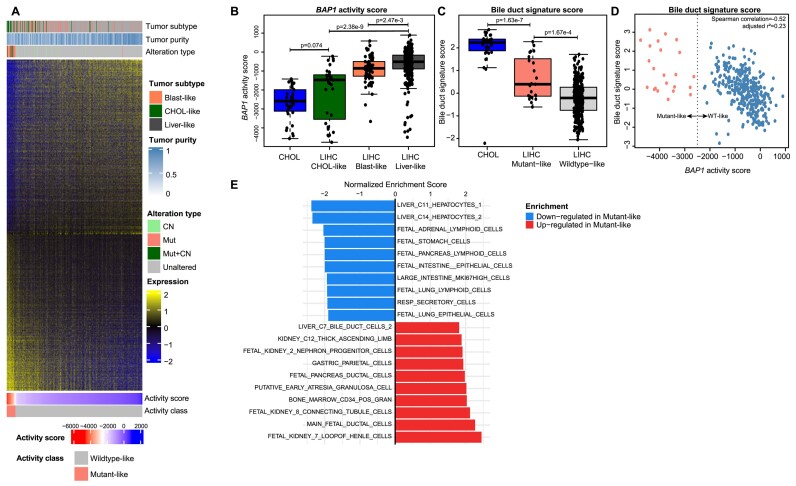
*BAP1* mutant-like hepatocellular carcinoma (LIHC) tumors are associated with a more duct-like phenotype. (**A**) Heatmap of differentially expressed genes between *BAP1* mutant-like and wildtype-like tumors in LIHC with sample annotations. CN: gene-level copy number loss; Mut: mutation only; Mut + CN: mutation and gene-level copy number loss. (**B**) CHOL and LIHC *BAP1* activity scores separated by molecular subtype from (13). *P*-values are derived from pairwise two-sided Mann–Whitney *U* tests with continuity correction and Bonferroni adjustment. (**C**) Specific comparison between CHOL and LIHC *BAP1* mutant-like and wildtype-like samples of bile duct gene expression from four combined signatures from the MSigDB C8 gene set collection. Adjusted *P*-values are derived from pairwise two-sided Mann–Whitney *U* tests with continuity correction and Bonferroni adjustment. (**D**) Scatterplot of *BAP1* activity and bile duct signature scores with indicated Spearman correlation; linear regression of *BAP1* activity and bile duct signature scores with Wherry adjusted *r*^2^ value is also shown. Dotted line represents classification threshold based on *BAP1*activity score. Points to the left of the dotted line indicate *BAP1* mutant-like samples and points to the right indicate *BAP1* wildtype-like samples. (**E**) Top 10 differential cell type signatures from the MSigDB C8 gene set collection in both directions between *BAP1* mutant-like and wildtype-like LIHC tumors. Shown results are statistically significant with Benjamini–Hochberg adjustment. Lower, right side-oriented bars: upregulated in *BAP1* mutant-like samples; upper, left side-oriented bars: downregulated in *BAP1* mutant-like samples.

Because *BAP1* alterations have been previously shown to constitute a molecularly distinct subset of tumors in LIHC and may play a unique role in regulating cell identity and differentiation processes in the liver, we selected LIHC for more focused analyses (13,50–52). In LIHC, *BAP1* mutant-like tumors were distinct from wildtype-like tumors (Figure 4A). Annotations of tumor molecular subtyping from Damrauer *et al.* revealed that the *BAP1-*altered subset was enriched for blast-like and CHOL-like LIHC samples (13). Consistent with these previous data, 17/31 (54.8%) of *BAP1*-altered tumors were one of these two molecular subtypes; among the unaltered tumors, 77/327 (23.5%) were one of these subtypes. Blast-like and CHOL-like tumors have *BAP1* activity scores lower than the other LIHC tumors (pairwise two-sided Mann–Whitney *U* test with continuity correction and Bonferroni adjusted *P*= 2.47e−3 and *P*= 2.38e−9, respectively; Figure 4A and B, and Supplementary Table S3B).

We previously hypothesized that the CHOL-like subset arose due to de-differentiation (13). To further explore the relationship between *BAP1* and less-differentiated (or more CHOL-like) LIHC tumors, we examined expression of bile duct cell markers based on four combined bile duct cell signature gene sets. We used true CHOL samples as a comparison and observed that *BAP1* mutant-like LIHC tumors were enriched for bile duct markers relative to wildtype-like samples (pairwise two-sided Mann–Whitney *U* test with continuity correction and Bonferroni adjusted *P*= 1.67e−4; Figure 4C). Further, bile duct signature scores in LIHC tumors had a moderate negative correlation to *BAP1* activity scores (Spearman correlation = −0.52; Figure 4D), suggesting that the signature captures expression of some genes that separate cholangiocyte and hepatocyte identity. A broader analysis of cell type signatures showed enrichment of bile duct and progenitor markers and a decrease in terminally differentiated cell markers, for example hepatocyte markers (Figure 4E), consistent with a transcriptional profile shift toward a less- or transdifferentiated phenotype.

### Association of *BAP1* with developmental timing

**Figure 5.**
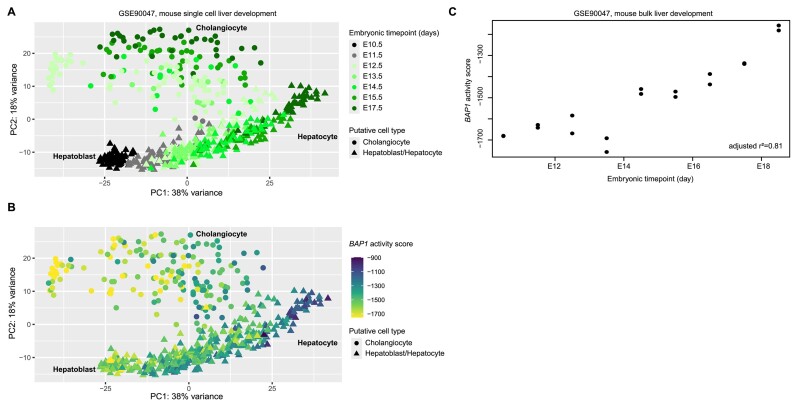
Earlier timepoints in mouse embryonic liver development are associated with lower *BAP1* activity scores. (**A**) Principal component analysis of single-cell RNA-seq data demonstrating temporal separation of putative hepatoblast, hepatocyte and cholangiocyte cell types, colored by embryonic timepoint. (**B**) Principal component analysis of the same single-cell dataset as in panel (A), colored by *BAP1* activity score. (**C**) Scatterplot of embryonic timepoint and *BAP1* activity score in a bulk RNA-seq dataset for mouse liver embryogenesis. Statistic is linear regression with Wherry adjusted *r*^2^.

To further understand the potential role of *BAP1* in liver development and cellular differentiation, we explored temporal changes in *BAP1* activity scores within an external single-cell RNA sequencing (RNA-seq) dataset of mouse embryonic liver development (GSE90047) (53). Principal component analysis of single-cell data demonstrates separation of cells both by embryonic timepoint and by putative cell type along the first two principal components (Figure 5A). When *BAP1* activity score values were overlaid on top of these data, there was a clear trend of lower scores (more mutant-like) in cells that are earlier in development; as the embryonic timepoint increases toward a more differentiated phenotype, scores increase along a pronounced gradient (Figure 5B). This trend holds in bulk RNA-seq data from the same study, showing a high correlation between embryonic timepoint and *BAP1* activity score (adjusted *r*^2^= 0.81; Figure 5C).

## Discussion

### *BAP1* alterations are frequent in cancer

Here we report a 41% increase in detection of *BAP1* somatic mutations across TCGA compared to prior mutation calls (32) by using a *de novo* local realigner and merging variant calls from six individual callers across two pipelines. While long-read sequencing technology has become more accessible and has improved detection of larger indels, historical datasets such as TCGA with short-read sequencing will continue to benefit from reanalysis as genome builds and gene models evolve and newer and improved tools capable of detecting additional variants are developed. Importantly, we were able to identify additional indels ≥40 bp that previously were undetected. While long-read sequencing technology has become more accessible and has improved detection of larger indels, historical datasets such as TCGA with short-read sequencing will continue to benefit from reanalysis using newer and improved tools capable of detecting additional variants. Our work also makes available an efficient variant calling pipeline that can be easily and readily applied to other cancer genes while substantially reducing file storage requirements and increasing computational speed through use of BAM slices and parallelization.

Copy number loss of *BAP1* is found in ∼20% of TCGA pan-cancer samples, making it the most common alteration of *BAP1* in this cohort (25). However, chromosome 3 is home to multiple tumor suppressors important in many cancers (*VHL*, *SETD2*, *PBRM1*) in addition to *BAP1*, making it unclear in which cancers *BAP1* loss is biologically important. Using gene expression data from tumors with the highest frequency of *BAP1* mutations, we developed a score that could infer which alterations were likely affecting *BAP1* activity or function. We validated our score using data from *BAP1* knockout cancer organoid models that had lower *BAP1* activity scores compared to parental organoids. When we assessed tumors using this score, we found a subset of copy number loss events that were associated with reduced *BAP1* activity. Further, *BAP1* copy number loss in conjunction with *BAP1* mutations showed the strongest reduction in inferred *BAP1* activity or function.

### Role of *BAP1* in cell identity and cell state changes

*BAP1* is important in cell differentiation and development (50,54–56). Patel and Yanai have postulated that tumor plasticity is ‘constrained by the organism’s developmental map’ (57). For example, hepatocytes and cholangiocytes may both de-differentiate toward their shared progenitor phenotype in tumorigenesis. Our prior work identified a subset of pathologically defined hepatocellular carcinoma (CHOL-like) with mutations and gene expression profiles more similar to cholangiocarcinoma (13). These tumors were originally defined by *IDH1* loss and we further showed them to have *BAP1* loss as a major altered feature. In our current study, hepatocellular carcinoma tumors with low *BAP1* activity scores had higher expression of bile duct signatures. Additionally, the subset of CHOL-like hepatocellular carcinomas had lower *BAP1* activity scores and were phenotypically more similar to cholangiocarcinomas than to other hepatocellular carcinoma samples.

Our results from the TCGA cancer dataset suggest that loss of *BAP1* leads to dysregulation of mechanisms governing cell fate and consequently to changes in cell states. Because *BAP1* has well-known roles in normal development (54), we assessed changes in *BAP1* activity scores during normal mouse liver development. Using a single-cell RNA-seq mouse embryonic time course, we found that *BAP1* activity scores were low in progenitor cells and increased during liver development (53). These results are consistent with *BAP1*’s known role as an important coordinator in differentiation processes and maintenance of cell identity and support the hypothesis that *BAP1* dysregulation in somatic cells can lead to cell states resembling earlier stages of development in the liver cell lineage.

## Supplementary Material

zcae045_Supplemental_Files

## Data Availability

Whole exome sequencing BAM files are available by controlled access through the NIH Database of Genotypes and Phenotypes (dbGaP, https://www.ncbi.nlm.nih.gov/gap/). Supplementary Table S1 contains a list of case and file IDs downloaded from the NIH Genome Data Commons repository (https://portal.gdc.cancer.gov/repository). The following data were downloaded from the TCGA PanCanAtlas Publications webpage (https://gdc.cancer.gov/about-data/publications/pancanatlas): Pan-cancer variants MAF: mc3.v0.2.8.PUBLIC.maf.gz ABSOLUTE purity/ploidy file: TCGA_mastercalls.abs_tables_JSedit.fixed.txt TCGA Clinical Data Resource (CDR) Outcome: TCGA-CDR-SupplementalTableS1.xlsx Tumor subtype annotations of TCGA samples were compiled from many published sources. PubMed Central identifiers are available in Supplementary Table S3. Human bulk liver organoid RNA-seq data (GSE215785 and GSE129457) and mouse bulk and single-cell liver developmental RNA-seq data (GSE90047) were downloaded from the Gene Expression Omnibus repository (50,51,53). Nextflow code, BED files and a Singularity container of software used for variant calling with ABRA2/Cadabra/Strelka2 are available on Zenodo (DOI: 10.5281/zenodo.10175692, https://zenodo.org/doi/10.5281/zenodo.10175692). R code for generating all figures using data from the supplemental tables is also available on both Zenodo and the GitHub project repository for this manuscript (https://github.com/isturgill/Sturgill_BAP1).
