# Supplementary material for: Expanded detection and impact of BAP1 alterations in cancer
Source: NAR Cancer. 2024 Nov 15;6(4):zcae045. doi: 10.1093/narcan/zcae045 (PMC11567159; doi:10.1093/narcan/zcae045)
Supplement: zcae045_Supplemental_Files [file zcae045_supplemental_files.zip › supplemental_figures_merged.pdf]

Supplemental Figure S1

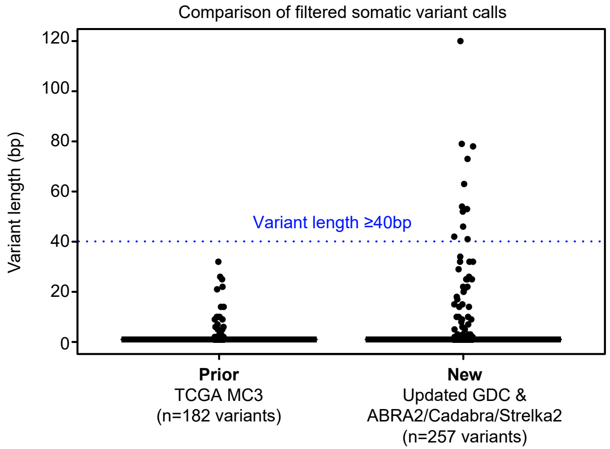

Supplemental Figure S2

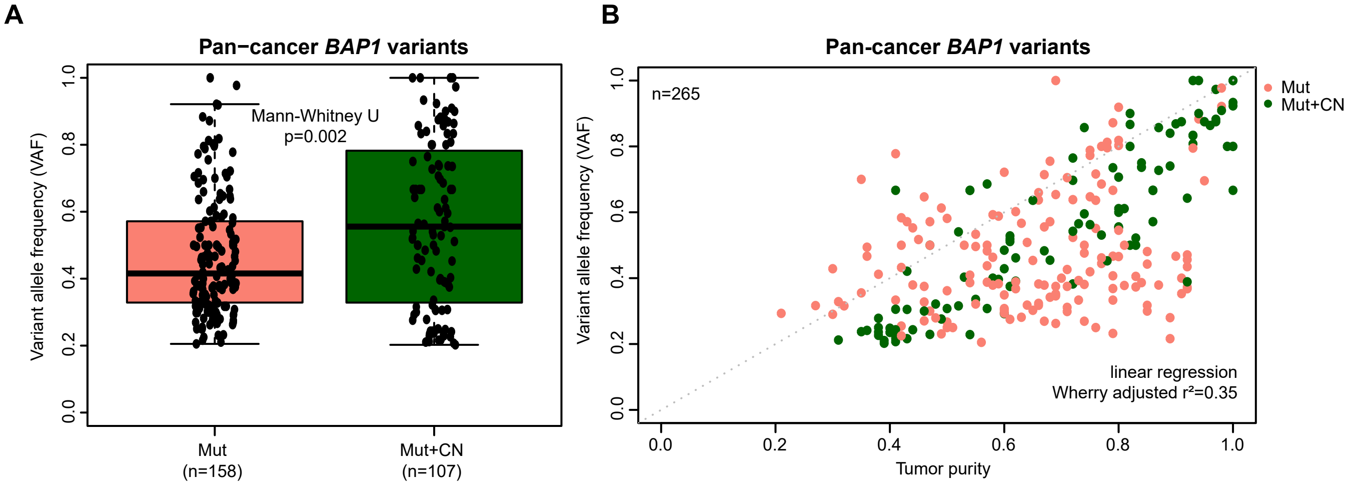

Supplemental Figure S3

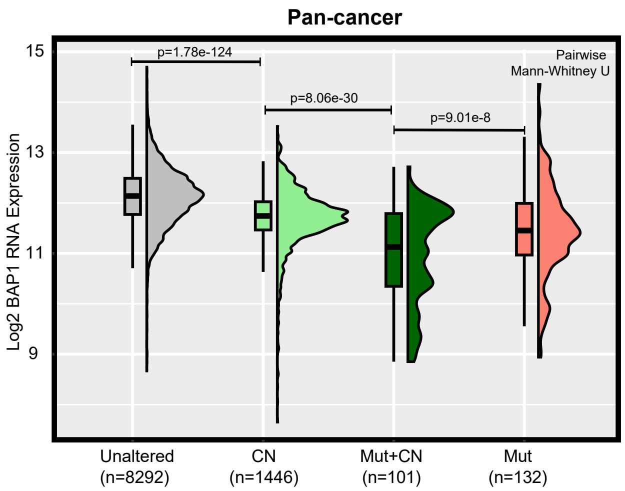

Supplemental Figure S4

A

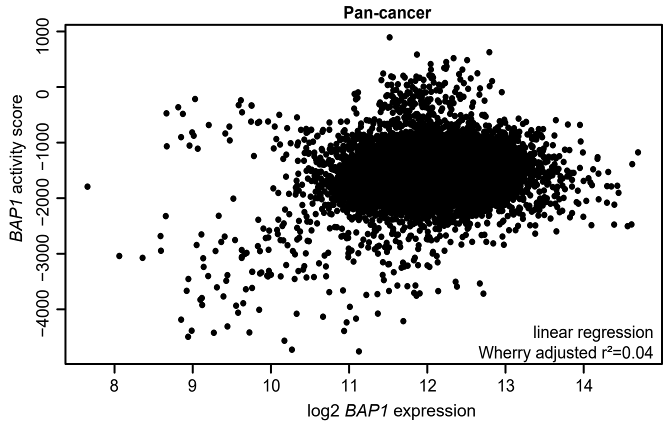

B

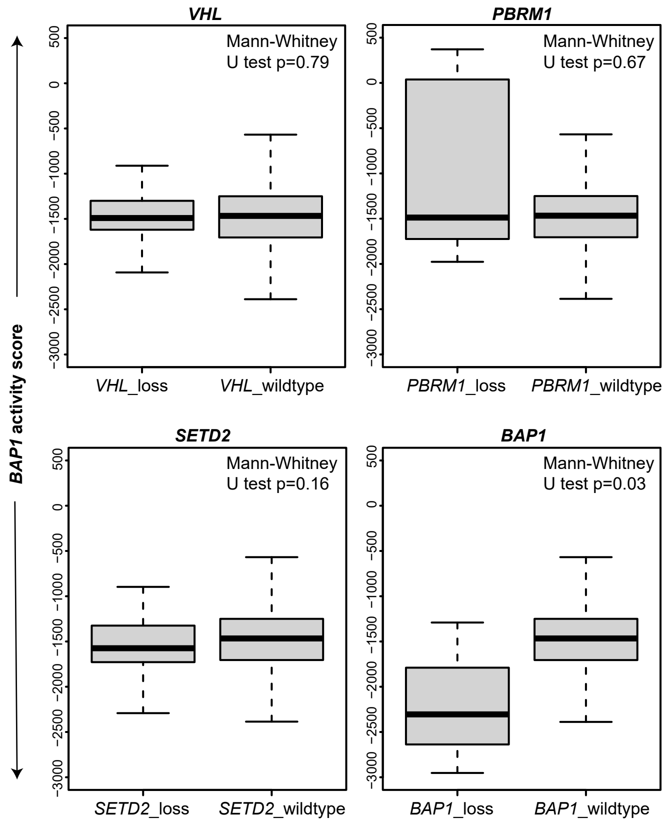

Supplemental Figure S5

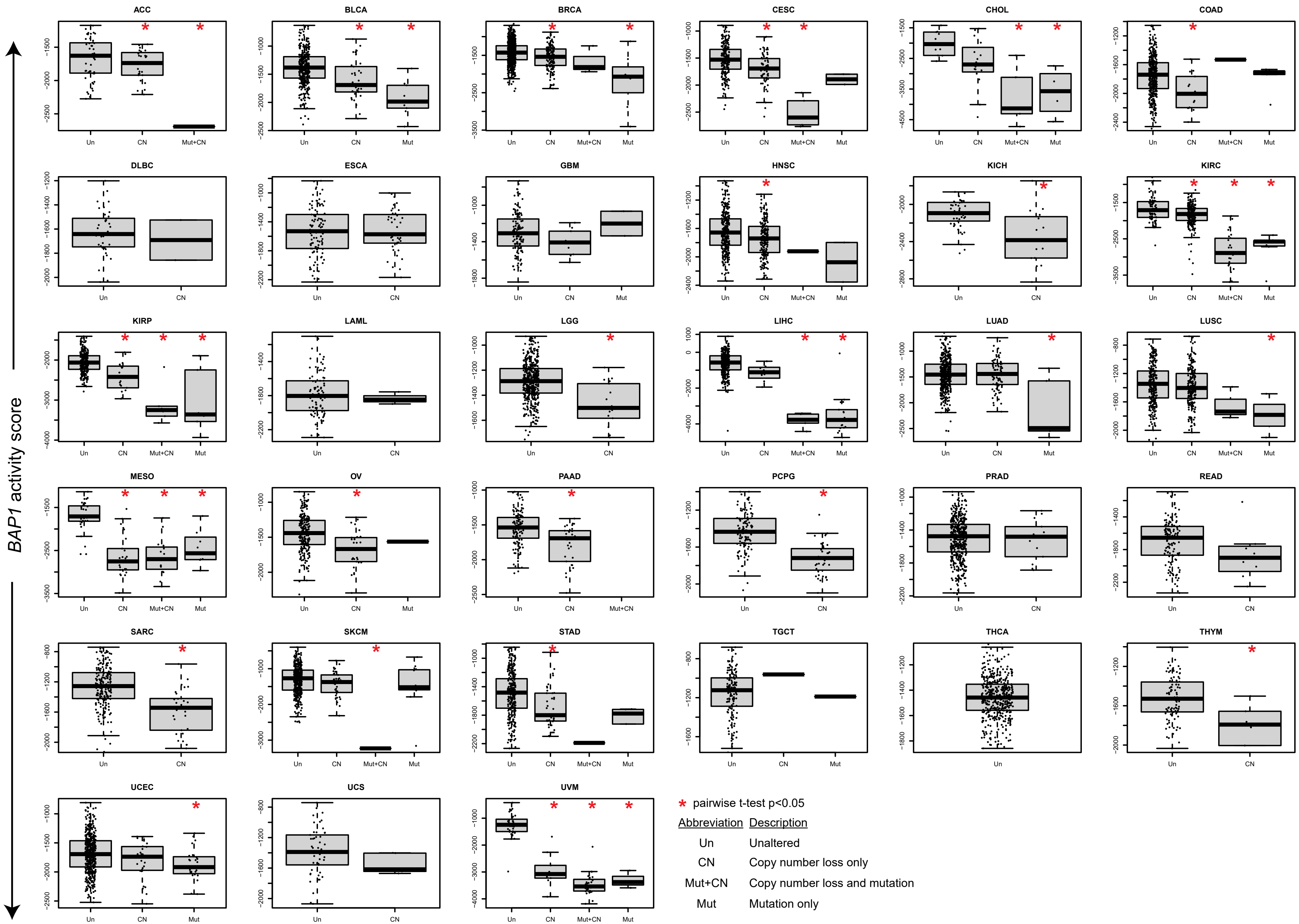

Supplemental Figure S6

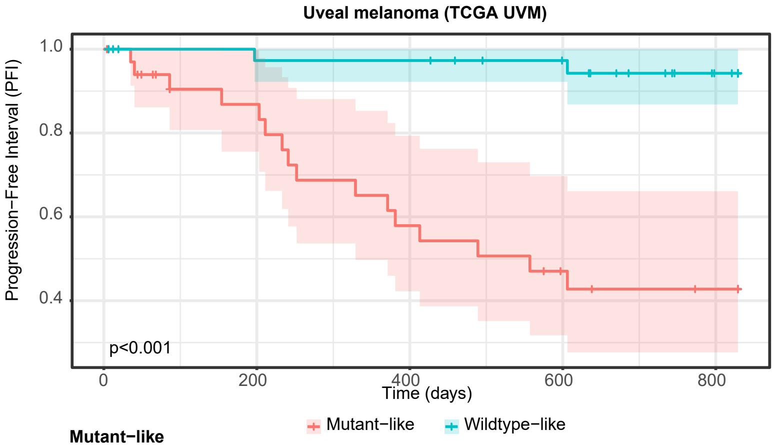

	0	200	400	600	800
**Mutant−like**					
At Risk	34	24	16	11	8
Events	0	4	12	15	16
**Wildtype−like**					
At Risk	41	36	36	32	22
Events	0	1	1	1	2

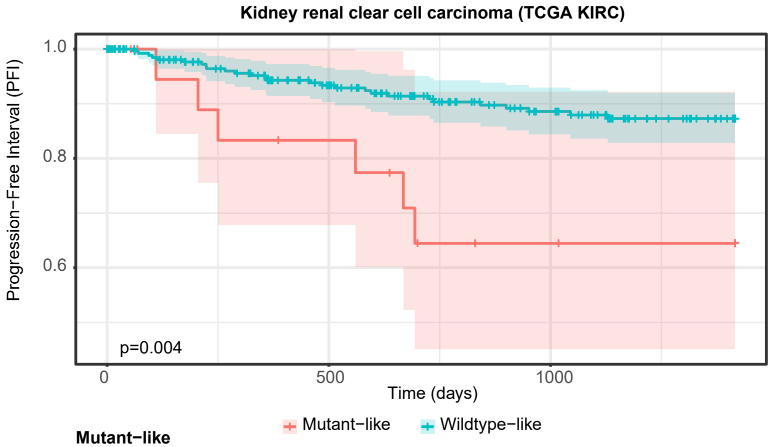

	0	500	1000
**Mutant−like**			
At Risk	21	14	8
Events	0	3	6
**Wildtype−like**			
At Risk	272	199	145
Events	0	16	25
