# Supplementary material for: Expanded detection and impact of BAP1 alterations in cancer
Source: NAR Cancer. 2024 Nov 15;6(4):zcae045. doi: 10.1093/narcan/zcae045 (PMC11567159; doi:10.1093/narcan/zcae045)
Supplement: zcae045_Supplemental_Files [file zcae045_supplemental_files.zip › supplemental_figure_legends.docx]

Supplemental Figures

Supplemental Figure S1. *BAP1* variant lengths for variants detected across mutation calling pipelines, comparing the prior TCGA MC3 dataset (n=182 variants) and the new dataset (n=257 variants) which combined updated TCGA GDC variant calls with calls from an ABRA2/Cadabra/Strelka2 workflow. Blue dotted line indicates the threshold for variant lengths ≥40bp.

Supplemental Figure S2. **(A)** Variant allele frequency for *BAP1* variants in pan-cancer mutant samples, separated by alteration type. P-value derived from two-sided Mann-Whitney U test with continuity correction. **(B)** Scatterplot of tumor purity and variant allele frequency for *BAP1* variants in pan-cancer mutant samples, colored by sample alteration type. Dotted line represents identity (y=x) line. Adjusted r-squared value derived from linear regression in R with Wherry adjustment. Mut: mutation only, Mut+CN: mutation and gene-level copy number loss.

Supplemental Figure S3. Pan-cancer *BAP1* RNA-level expression by alteration type. P-values derived from pairwise two-sided Mann-Whitney U test with continuity correction and Bonferroni adjustment. CN: gene-level copy number loss only, Mut: mutation only, Mut+CN: mutation and gene-level copy number loss.

Supplemental Figure S4. **(A)** Pan-cancer scatterplot of tumor *BAP1* RNA expression and *BAP1* activity score. Adjusted r-squared value derived from linear regression in R with Wherry adjustment. **(B)** Comparison of *BAP1* activity scores by chromosome 3p gene copy number status (*VHL*, *PBRM1*, *SETD2*, and *BAP1*). P-values derived from Mann-Whitney U tests with continuity correction and Bonferroni adjustment.

Supplemental Figure S5. Per-cancer boxplots of *BAP1* activity scores by alteration type. Un: unaltered, CN: gene-level copy number loss only, Mut+CN: mutation and gene-level copy number loss, Mut: mutation only. Red asterisk indicates statistical significance with p<0.05 from pairwise two-sided t-test with Bonferroni adjustment.

Supplemental Figure S6. Kaplan-Meier progression-free survival curves for TCGA UVM and TCGA KIRC tumor types, stratified by *BAP1* mutant-like or wildtype-like classification. P-values are derived from the log rank test in univariate analysis. Shaded backgrounds for each curve represent the 95% confidence interval.
